# Supplementary material for: A Qualitative Investigation of the Psychosocial Impact of Chronic Low Back Pain in Ghana
Source: BMJ Open. 2023 Jul 20;13(7):e073538. doi: 10.1136/bmjopen-2023-073538 (PMC10360413; doi:10.1136/bmjopen-2023-073538)
Supplement: Supplementary data [file bmjopen-2023-073538supp001.pdf]

Supplemental File 1: Categories, Concepts, Codes, Mechanisms & Exemplar Quotes

Categories	Concepts	Codes	Mechanisms	Exemplar Quotes
Loss of Self & Roles	Loss of Self<br>Loss of Roles	➢ Unpleasant experience/ Unbearable pain<br>➢ Unplanned Change<br>• Loss of spontaneity/ referred pain/stiffness/ altered posture.<br>• Interference with conjugal relationships/ family life.<br>• New Self<br>• Stopped/ Altered domestic, work, gendered, cultural, social activities and hobbies.<br>➢ Time constraints	**Predominant Conditions/Consequences:** Personal experiences, cultural influences, socio-economic impact, patients & HCPs' bio-medical/mechanical beliefs<br>**Others:** Multiple hospital visits, Inadequate CLBP education<br><br>**Derived Mechanisms:** HCPs' and patients' bio-medical/mechanical beliefs, sociocultural beliefs, socioeconomic impact	*"I cannot even do household work. You see as a man sometimes I live with a wife…you have to do some scrubbing, some house-chores to help the lady. I have been advised by doctors… don't bend so I am careful not to do the dos and donts" (P6S1).*<br>*"As a matter of fact, when it started, the pain was so severe that…I had to stop gyming too and it is a hobby. I used to do it for fitness too because of my job" (P13S2).*<br>*"…it has been there for long. I have suffered. Sometimes I cannot even sleep. My eyes are open all night. Now that the weather is cold, it's not easy. When I wake up It's very painful. I have become a different person" (P11S1).*<br>*"I couldn't do anything. I couldn't sleep. I used to go and help tidy up the church. But since I got ill for about two years now, I am not able to go to church … and walking a lot makes me tired. So as for this sickness, it has changed every aspect. I used to try to wash my clothes and do things, but when I came here (hospital) they advised me to stop… they made me understand I am doing a lot of wrong things" (P2S2).*<br>*"Your sexual performance too, it also affects your sexual performance…I took the X-ray. So, I was advised that if I don't treat it well, it would be on and off so I was advised that I should stay away from sex for now. I decided to do it that way" (P13S2).*<br>*"Waist pain is something that when it happens to you, you cannot do anything. You cannot work, the pain it's too much" (P11S1).*<br>*"Yes, it has affected me. Erm I'm a quick person who does things quickly but now it has reduced my speed in doing things. Even the walking. It has reduced my walking, erm house-chores even itself I can't do it much because if I continue doing it much then I start having the pain" (P9S1).*

				"I have changed…when I sit, I can't get up as if I had been glued. When you wake up, you cannot get up properly and it's so painful and you know then you cannot straighten up… after the scan they said they detected something small, and they gave me some drugs…I don't have much of a social life because of the pain" (P15S1)<br>*"I was not able to be active like at first. At first, I was smart. But now I'm like dull" (P3S2).*<br>*"Since it started it has affected my work and everything I do. I don't get time.. I can't work. When I sit for a while, it hurts. And also I have to come to the hospital and I have a baby so even with washing and all that I suffer" (P3S1).*<br>*"It's very painful. Severe pain. I cannot do my house chores like before. It's not easy. I cannot sand for long and at work I have to manage" (P1S1)*<br>*"Me, my first born is 13years. It's the small one who is four years, so when I am taking him to school, I have to carry him. Because I'm in a hurry I put him at my back and board a car with him. That has become difficult for me now, because the doctors said carrying heavy things is not good for me" (P7S2).*
Fear of the Future	Fear of disability & death.<br><br>Fear of toxicity, side-effects associated with orthodox medication use.	• Premature ageing<br>• Chronicity<br>• Stigma with assistive devices<br>• Fear of paralysis<br>• Side effects of medication<br>• Cautious about simultaneous use of herbal and orthodox medications	**Predominant Conditions/Interactions:** HCPs' biomedically oriented education/advice/treatment, sociocultural beliefs.<br>**Others**- Pain Response/Personal Experience, Inadequate Patient Education, Internet sources and media.<br><br>**Derived Mechanisms:** Sociocultural beliefs,	*"I don't ever want to be walking with stick…it makes you look old. I should use walking aid after 100 (laughs)" (P1S2).*<br>*"Even initially walking was very difficult for me. But I was struggling. I decided not to use any of those things. I thought I could become old all of a sudden, premature aha ageing. Aha so I was struggling. Somewhere not to walk koraa I was struggling. I would just put my hands round, and in the middle, that was it" (P12S1).*<br>*"Things I have read on the internet. They said it wouldn't kill you, but you would be paralyzed, that's what they said" (P9S2).*<br>*"So, I was asking myself is that how I'm going to be for the rest of my life? They (HCPs) telling you don't drive don't do this don't do that. I fear I would not be able to move around freely" (P14S2).*<br>*"Yes I take herbs. Hospital medication, I find out that my stomach; then people were advising me that the chemicals are too much…*

		• Cautious of orthodox medications	patients' & HCPs' bio-medical/mechanical beliefs.	*they advise that the herbs doesn't contain so much chemical so…" (P13S1).*<br>*"the herbs some you rub on the back so it does not affect the stomach like that. The medications, painkillers there are side-effects so the doctors tell you don't take it all the time…that's another worry" (P8S1).*<br>*"Everybody wants life with strength. As for life, if you are alive and you cannot lift your hand to your mouth, it is not beneficial. So, if the time is not yet up and you are able to move, you accept it that way and do as they (HCPs) say so you don't become disabled. I am grown, next year July, I would be 70. The time is not yet up and then you have become something else (disabled), then it means even the children would neglect you." (P2S1).*
Emotional Distress	Feeling of unhappiness & anger<br>Frustration & Suicidal Ideations	• Crying, not happy<br>• Helpless<br>• Frustrated<br>• Withdrawal<br>• Thinking about the pain<br>• Suicidal Ideations	**Predominant Conditions/Consequences:** Personal experiences, cultural influences, socio-economic impact, patients & HCPs' bio-medical/mechanical beliefs<br>**Others:** Multiple hospital visits<br><br>**Derived Mechanisms:** HCPs' and patients' bio-medical/mechanical beliefs, sociocultural beliefs, socioeconomic impact	*"It has really worried me. If I don't say it, I'd be a liar (cries). I cannot go to church, I cannot work, no money, at some point I could not walk" (P2S2).*<br>*"Doing anything...those times you walk from the entrance to the fevers unit. The pain that I go through I even cry" (P6S2).*<br>*"The mind doesn't think properly. Then it's like anybody you see is an enemy. It becomes sensational" (P1S2).*<br>*"I was helpless and sometimes I'd be in the house and will be thinking why can't I do the things I used to do before…and sometimes I weep. I have lost my work contacts. I stopped lifting heavy things, sitting for too long because the doctors and physios have advised that these activities don't help" (P7S2).*<br>*"I felt helpless" (P14S1).*<br>*"Everything. Me I'm tired o. I am not myself. Hospital, the pain, everything. Because I'd be coming all the way…and buy this medication, do this do that ...I am tired…(sobs)" (P9S2).*<br>*"My mother in-law has that chronic thing. She's not taking it easy at all, And I started crying. So, I mean it's not just easy for those who have that chronic one, managing it is not just easy" (P14S2).*

				"As an elderly woman you cannot even perform your duties. It makes me sad. I have been advised to stop bending, standing and walking for long they (HCPs) say it's not good, so I don't do it. Sometimes too when I stand for long I get pain too" (P11S1).
Stigmatization & Marginalization	Stigmatization Marginalization	• Marginalization at work and home<br>• Stigma at the household, work and societal level	**Predominant Conditions/Consequences/ Interactions:** Cultural influences, socio-economic impact, patients & HCPs' bio-medical/mechanical beliefs.<br><br>**Derived Mechanisms:** HCPs' and patients' bio-medical/mechanical beliefs, sociocultural beliefs, socioeconomic impact	*"My job. I can tell you they even sent me to EOCO. Because they said auditors came, they audited me and I was supposed to answer some questions, I didn't avail myself. Meanwhile I had all this medical report but suddenly hearing that none of my medical report are found on my file. And when it came like that, they were just throwing the rumour that it's true that I have squandered money that is why, meanwhile I was in hospital" (P13S1).*<br>*"With the pain, now I don't have much of a social life. When you go out you might end up restricting everyone and it's not everything you can do and you cannot keep on explaining. People keep complaining that you have changed, your character has changed so I decided to keep to myself. I concentrate more on fitness now" (P15S1).*<br>*"The happiness in the house has been affected. My husband feels I have changed because of the pain. He doesn't know what I am going through so he ignores me…but I also feel I need to protect myself by avoiding sex and other things…there is no joy" (P8S2).*<br>*"Sometimes they think you are pretending…I remember one morning I couldn't get up. I called my husband and told him I cannot get up he thought I was lying" (P1S1).*<br>*"When you are bent and walking around people stare at you. I have been told the corset would help straighten me up" (P4S2).*<br>*"Sometimes when colleagues and supervisors at work see you taking lots of break to stretch, it becomes something else. The doctor and physio advised that. My boss was complaining because now I cannot push myself like before" (P3S2).*
Social Support	Family & Friends Support	• Family and friends as prescribers	**Predominant Conditions/Interactions/ Consequences:** Socio-	*"My child there is no money. It (CLBP) has brought difficulties. I am not able to work" (P11S1).*

	Financial Burden	• Family and friends support with coping<br>• Lack of social support/work welfare<br>• Financial constraints (lack of funds & increased expenditure)	cultural influences, Socio-economic impact, HCPs biomedical orientation<br><br>**Derived Mechanisms:** Sociocultural beliefs, patients' & HCPs' bio-medical/mechanical beliefs.	*"Since the pain about a year ago, I've been in in the house doing nothing. The MRI showed that the bones have pulled out. The doctors said they would help me for it to go back to its normal state. I had to mobilize funds and now my income has reduced" (P7S1).*<br>*Economically I am down. Sometimes I have to beg from my friends and I am supposed to be the breadwinner for my family, my extended family too" (P10S1).*<br>*"Even your finances. You might not even be able to drive your car properly, you have to get someone to drive. In fact so completely it derails you. It increases your financial burden" (P1S2).*<br>*"And walking a lot makes me tired. It makes me tired. And because of it I cannot do any hardwork, It has affected my finances. The doctors too they've made us understand that some things that we do like bending, lifting, sitting too long we should not do it. It makes the condition worse" (P2S2).*<br>*"…so it was later someone suggested helping me. My children, my children are there. If I have to go to the toilet, they help me. But since I was given the belt, now I don't have to hold the rails anymore. I walk myself. But I am not able to walk fast however I do it bit by bit". (P11S1)*<br>*"My children, they feel they have to take care of me. Mmm sometimes, they asked me not to be doing it but I've been doing it. Like. They asked me not to be lifting too much heavy things, I shouldn't be washing. Even if its paining me I do bear the pain and last a friend asked me to buy this…we have a spray medication. (Hisses) so I bought it. But once a while when it's paining me in the morning and I don't want to take any medication, then I just spray a little at the back" (P9S1).*<br>*"And I attend church with one Bro. E. So, I discussed it with him. Then he suggested that I come for physiotherapy and see" (P14S1).*<br>*"I play with my children a lot, so it helps me cope with the pain" (P8S1).*

				"With the house chores too, my children are there so I don't really do a lot of work at home" (P12 Site1).<br>*"Yes, my wife has been very much supportive. My Christian brothers have also been supportive but not my extended family" (P14S2).*<br>*"Like the local medicine, one day I went to the market and when I was walking, I bent down. So, when I bent, a woman asked me why and I told her. She said some few days ago something like that happened, so I should get some local spices. I did it" (P5 Site1).*

HCPs- Healthcare Professionals. Participants mainly referred to doctors and physiotherapists when HCPs/doctors were used in their narrations.
